# Study of ultrastructure and apoptosis in the endometrium of women with or without endometriosis

**Published:** 2013-05

**Authors:** Leila Roshangar, Seddighe Abdollahifard, Abbas Majdi, Armin Zarrintan, Alia Ghasemzade, Laaia Farzadi, Sara Soleimani Rad, Jafar Soleimani Rad

**Affiliations:** 1 *Neuroscience Research Center and Drug Applied Research Center, Tabriz University of Medical Sciences, Tabriz, Iran. *; 2 *Alzahra Hospital, Faculty of Medicine, Tabriz University of Medical Sciences, Tabriz, Iran.*; 3 *Department of Anatomical Sciences, Faculty of Medicine, Tabriz University of Medical Sciences, Tabriz, Iran.*; 4 *Department of Histology and Embryology, Faculty of Medicine, Tabriz University of Medical Sciences, Tabriz, Iran.*

**Keywords:** *Endometriosis*, *Endometrium*, *Stroma*, *Ultrastructure*, *Apoptosis*

## Abstract

**Background: **More than 40% of infertilities are due to endometriosis. Ultrustructural and histochemical study of endometrium will help to clarify the etiology of endometriosis.

**Objective:** The aim of the present study was to investigate the ultrastructure and occurrence of apoptosis in endometrial cells of women with or without endometriosis.

**Materials and Methods: **In the present case-control study, endometrial specimens from 12 women without endometriosis (as control) and 12 women with endometriosis (as case) were examined. Specimens for control group were obtained from the patients that were referred to gynecology hospital for hysterectomy due to various reasons. In case group the endometriosis was diagnosed according to laparoscopy and endometrial samples were taken using pippel biopsy. The specimens from both case and control groups were processed for Transmission Electron Microscopy (TEM), TUNEL reaction technique and morphometric studies.

**Results:** The results show that endometrial epithelium lost its continuity in women with endometriosis and endometrial cells have euchromatic nucleus in comparison to those from non-endometriosis. There were several apoptotic cells in the luminal and glandular endometrial epithelium and stroma from endometrium of control group. However, apoptotic cells were rarely seen in the endometrium from women with endometriosis. The difference in number of apoptotic cells between two groups statically was significant (p<0.001).

**Conclusion:** Regarding the ultrastructural characteristics of endometrial epithelial cells and comparison of apoptotic occurrence in control and case groups it is concluded that endometrial cells in endometriosis group have higher potential to survive and possibly implant.

## Introduction

Endometriosis is defined by the presence of endometrial tissue at locations outside the uterus. Endometriosis, a disease affecting 10% of women of reproductive age, is one of the most frequently encountered gynecological disorders that its pathogenesis is poorly understood. As such, it is one of the most common causes of infertility, dysmenorrhea and pelvic pain (1). Endometriosis is also the cause of infertility in more than 40% of infertile women (2). 

The proposed theories on pathogenesis of endometriosis are not conclusive and endometriosis should be considered a disease with multiple etiologies (3-7). However, the most accepted theory is still the theory of Sampson or menstrual reflux hypothesis (3). According to this hypothesis during menstruation, endometrial cells move through the fallopian tubes in a retrograde manner and implant to the pelvis and other abdominal organs and continue proliferation. 

It should be noticed that retrograde menstruation and endometrial cells delivery may occure in all women and could be considered as a physiological phenomenon. The exception is that: in an individual with endometriosis the misplaced endometrial cells, for some reason, could survive and implant (4, 5, 8). The factors, which facilitate survival and implantation of misplaced endometrial cells, may contribute to the development of endometriosis. These mechanisms are necessary but insufficient to explain why only some patients develop the disease (6, 9-11). 

Apoptosis is a fundamental physiological process responsible for maintaining homeostasis in multicellular organisms, and play a critical role in maintaining tissue homeostasis and normal function and eliminating excess or dysfunctional cells. It is proposed that resistance to apoptosis and changes in the expression of some bioactive molecules are involved in the development of apoptosis (12-15). It appears that misplaced endometrial cells in women with endometriosis are metabolically active and facilitate their implantation and proliferation. 

On the other hand, ultrastructural characteristics such as size and extension of intracellular organelles are different in metabolically active and non-active cells. While there are very few studies indicating that ectopic endometrial tissue in endometriosis shows some differences, in comparison to eutopic endometrium in non-endometriosis (16). There is almost no comparative study on the ultrastructural characteristics of endometrial cells in endometriosis and non-endometriosis. Thus the aim of the present study is to investigate ultrastructural characteristics and occurrence of apoptosis in the endometrial luminal and glandular epithelium and stromal cells from women with endometriosis and comparing them with those from women without endometriosis. 

## Materials and methods

In the present case-control study, 12 women of reproductive age, without endometriosis, as control group, and 12 women of same age, with endometriosis as case group were examined. The samples were obtained from patients who referred to the university based Alzahra Hospital, Tabriz- Iran, during Jan 2007- Jan 2008 and were alleged for hysterectomy. In control group, endometrial specimens were collected from surgically removed uteri under sterile condition, put in a test tube containing PBS and then transferred to histology lab for further processing. In these patients it was scheduled that the surgeries to be carried out around mid-late secretory phase. Endometrial specimens were collected from those patients that visual inspecting, during surgery, showed no sign of endometriosis and other exclusion criteria include: the presence of malignant tumors, hyperstimulated patients and endometrial infection. 

The case group, were selected from the patients that had symptoms of endometriosis and their disease were confirmed by laparoscopic examination. Similar to control group in these patients the time of laparoscopy was scheduled to be carried out around mid-late secretory phase. In this group, specimens from ectopic endometrial tissue at grade 2 stage were collected from pelvis during laparoscopy and eutopic endometrium was collected by pippel biopsy. The specimens put in PBS and transferred to histology lab for further processing. The study was approved by Tabriz University of Medical Sciences Research Committee and financially supported by the Research affair Department based on a proposal leading to MSc. thesis. The Medical Ethics Committee of Tabriz University of Medical Sciences approved the research study. All participants were given adequate information and consent was obtained from each participant. 

**Transmission electron microscopy**

Half of the specimens were processed for transmission electron microscopy. The samples from patients with endometriosis patients or non-endometriosis were cut into pieces of 1×1 mm and fixed in 2% glutaraldehyde in a 0.1 M phosphate buffer (Thuringowa, Australia) and post fixed in 1% aqueous osmium tetroxide (TAAB, UK). The pieces were then dehydrated through graded concentration of ethanol, and embedded in resin. One micron semi-thin sections were stained with toluidine blue. Ultra-thin sections from selected blocks were stained with uranyl acetate and lead citrate and observed in a LEO 906 type transmission electron microscope (17). 

**Immunohistochemical study**

The other half of the specimens were used for determination of DNA fragments in apoptotic cells. For this purpose, the specimens were fixed in 10% neutral buffered formalin, embedded in paraffin and 3μm thick sections was processed for light microscopic studies. DNA damage was detected by use of an in situ cell death detection kit (POD, Roche laboratories, Germany) and TUNEL staining was carried out according to manufacturer’s instructions. For assessment of apoptotic cells the stained specimens were viewed under bright field microscope and TUNEL positive cells were detected on the basis of their brownish color and counted in 5 fields in each section. 

**Morphometric study**

Morphometric study was limited to the estimation of volume fraction of nucleous to the cytoplasm in the glandular and luminal epithelial cells. For this purpose, using Motic software system, the histological images of the endometrium were transferred to the monitor. Then a lattice of 20mm^2^ were superimposed on image and area of the nucleus and the cytoplasm were determined by point counting and volume fraction was obtained by dividing of nuclear value to cytoplasmic value. 

**Statistical analysis**

Statistical analysis was carried out using student t-test with the SPSS package to determine the significance of any differences seen in the parameters studied. The level of p<0.05 was considered as significant. 

## Results

The results are presented as EM studies, apoptosis assay and morphometric studies. 

**Electron microscopic studies**

**Figure 1:**
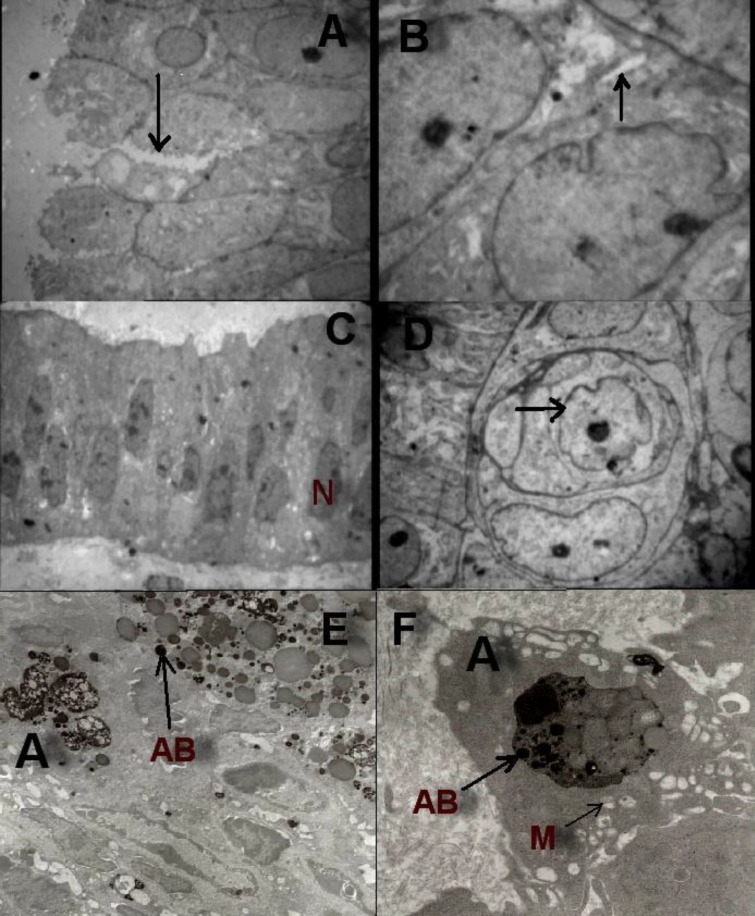
A, Electronmicrograph of endometrium of women with endometriosis showing disrupted areas (arrow) (X=1293); B, Electronmicrograph of apical part of luminal epithelium of women with endometriosis showing a cytoplasmic rupture (arrow) (X=3597); C, Electronmicrograph of luminal cells in case group which shows euchromatic and folded nucleous with volumous cytoplasm in luminal cells (X=1000); D, Electronmicrograph of stroma from endometriotic women showing abnormal cells (X=1670); E, Electronmicrograph of stroma of endometrium of control group demonstrating apoptotic cells (A) and apoptotic bodies (AB) in the stroma (X=1670); F, Electronmicrograph of stroma from control group demonstrating many ruptured and vacuolized mitochondria (M) and apoptotic cells (A) and apoptotic bodies (AB) (X=4646).

Electron microscopy revealed that endometrial luminal epithelium in the control group composed of regular columnar cells with heterochromatic nuclei (Figure 1C). Majority of mitochondria in this group appeared ruptured or vacuolized (Figure 1F). In addition, mitochondrial cristae were not clearly seen and most of them had been disappeared. Abundant dilated rough endoplasmic reticulum were present in many cells and the number of intracellular organelles was decreased. In comparison to control group in the case group the endometrial luminal epithelial cells has lost their continuity and numerous disruptions were present (Figure 1A). On the apical part of the endometrial luminal epithelium several ruptures in the cytoplasm were also present (Figure 1B). Most of the luminal cells in case group contained euchromatic and folded nuclei with volumous cytoplasm (Figure 1B). 

In the stroma from control group (women without endometriosis) there were several cells with characteristics of apoptosis (Figure 1E, 1F). That is, showing vacuolization, presence of apoptotic bodies, ruptured mitochondria, heterochromatic nuclei and nuclear blebbing. In the case group stroma contained some abnormal cells, appeared like a cell in cell structure and cellular nuclei were euchromatic (Figure 1D). 

**Apoptosis assay**

**Figure 2:**
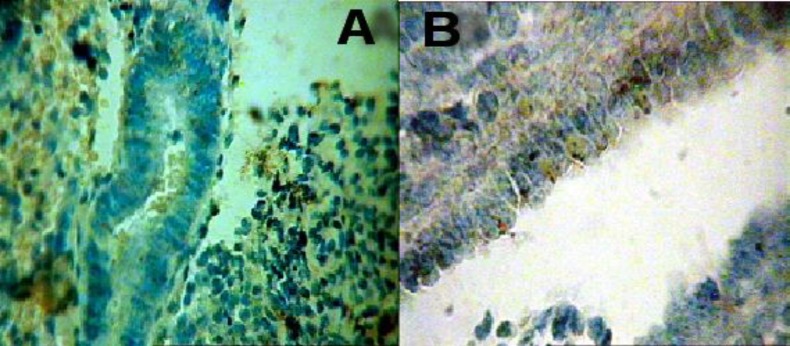
Photomicrographs showing TUNEL positive cells in the luminal and glandular epithelium and stroma of both case and control groups. A, Sectional photomicrograph from uterus of women with endometriosis at the middle of secretory phase, TUNEL positive cells are rare (X=40). B, Photomicrograph from uterus of control group in the middle of secretory phase, numerous TUNEL positive cells are present among epithelial and stromal cells (X=40).

Immunohistochemical studies for detection of apoptosis performed in paraffin sections using TUNEL technique. Brownish cells, representing TUNEL positive cells, were numerous in the epithetlial and or stromal cells from control group (Figure 2B). However, very few TUNEL positive cells were present in luminal and glandular epithelial and or stromal cells from case group (Figure 2A). 

Number of apoptotic cells in the epithelia and stroma are shown in table I. As it is shown in the table, the mean number of apoptotic cells in luminal and glandular epithelia, and stroma were significantly lower in the endometriosis group compared to the control group (p<0.001). 

**Morphometric studies**

In morphometric studies, the volume fraction of nuclei to cytoplasm in endometrial luminal and glandular epithelial cells is determined. The detailed data from morphometric are shown in table II. As it is shown in the table, volume fraction of nuclei to cytoplasm in both luminal and glandular epithelial cells were significantly (p<0.01) higher in the case group in comparison to control group. That is, 0.30±0.05 vs. 0.24±0.10 in luminal epithelial cells and 0.36±0.04 vs. 0.32±0.08 in glandular epithelial cells. 

**Table I:** Number of apoptotic cells in each sample from luminal and glandular epithelia and stroma (Mean±SEM) in control and case groups

	**Control group**	**Case group**
Luminal epithelium	1.05± 0.15	0.22± 0.10**
Glandular epithelium	1.11±0.21	0.16±0.09**
Stroma	16.16±0.54	7.45±0.80**

**Significant at p<0.001 (student t-test

**Table II:** Volume fraction of nuclei to cytoplasm in luminal and glandular ephitilial cells (Mean±SD) in control and case groups

	**Control group**	**Case group**
Luminal epithelial cells	0.24±0.20	0.30±0.05**
Glandular epithelial cells	0.32± 0.08	0.36± 0.04**

**Significant at p<0.01(student t-test

## Discussion

The results of the present study show that endometriosis has a negative effect on uterus, i.e., the ultrastructural changes in endometrial epithelial cells and occurrence of apoptosis in them were obviously different between case and control groups. Ultrastructural finding showed that endometrial epithelial cells has lost their continuity and appeared to be separating from neighboring cells. With regard to the reflux hypothesis and condition of endometrial epithelial cells, it can be proposed that in women with endometriosis, cellular transfer to pelvic cavity is facilitated (4, 18, 19). 

Furthermore, euchromatic nuclei of the endometrial epithelial cells and the increased volume fraction of the nuclei to cytoplasm are indicating that endometrial cells are more active in endometriosis than non-endometriosis. This is in support of the hypothesis that in women with endometriosis the endometrial cells have more potential to implant and proliferate (4, 20). 

Other ultrastructural findings include; vacuolization of luminal epithelial cytoplasm, mitochondrial vacuolization and disappearing of their cristae, presence of limited intracellular organelles and cystic shape of endoplasmic reticulum in the control group. These changes are evidences for suppression of cell potential for normal function and beginning of cell death process (21). 

In our study, ultrastructural findings well correlate with morphometric results, in this sense, endometrial cells in non-endometriosis have less potential to survive and probably to implant. Regarding relationship between morphological changes of mitochondria and cellular dysfunction, it is well shown that increased apoptosis rate and reduced ATP synthesis are associated with mitochondrial vacuolization and dissolution of their cristae. Vacuolization of mitochondria within the luminal and glandular epithelial cells may lead to weak capacity of cell survival and consequently causes cell death and prevent implantation of disseminated cells (22). 

Another finding of our study is that the numbers of apoptotic cells were decreased in endometriosis in comparison to non-endometriosis which again is in accordance with ultrastructural and morphometric results. This finding is in support of previous reports that showed endometrial cell apoptosis is decreased in euotpic and ectopic endometrial cell in women with endometriosis (8, 23). 

Apoptosis is responsible for balancing cell proliferation with cell death and for maintaining constant cell population in organs (24, 25). Approximately, 10% of epithelial and glandular epithelial cells undergo apoptosis during their development and proliferation (26). It has been indicated that activator of caspase-3, decreased luminal and glandular epithelial endometrial cells in women with endometriosis (27). This may indicate increased viability of endometrial cells shed during menses, facilitating their ectopic survival and implantation (8, 26-29). 

The present study also showed that apoptotic cells were less numerous in stroma from endometrium of case group and some stromal cells have atypical morphology (Figure 1D). There are evidences that endometrium possesses stem cells that are not only involved in endometrial regeneration and differentiation but also are involved in pathogenesis of endometriosis (30-32). 

## Conclusion

According to ultrastructural characteristic of luminal and glandular endometrial epithelial cells and the decreased apoptosis in endometriosis it is concluded that endometrial cells in control group have less chance of survival and or implantation. Conversely, in women with endometriosis they have higher potential for survival, proliferation and probably implantation. 
